# Culture-Negative Endocarditis and Cerebral Demyelination: A Challenging Case of Whipple’s Disease

**DOI:** 10.7759/cureus.32932

**Published:** 2022-12-25

**Authors:** Ana De Matos Valadas, Narcisa Fatela, Tiago Oliveira, Tiago Sepúlveda Santos, Maria Leonor Carvalho

**Affiliations:** 1 Internal Medicine, Centro Hospitalar Universitário Lisboa Norte, Lisbon, PRT; 2 Gastroenterology, Centro Hospitalar Universitário Lisboa Norte, Lisbon, PRT; 3 Pathology, Centro Hospitalar Universitário Lisboa Norte, Lisbon, PRT

**Keywords:** demyelination, clinical case report, culture negative infective endocarditis, whipple's disease, chronic diarrhoea

## Abstract

Whipple’s disease is a rare condition that, when not recognized and properly treated, can be fatal. A 49-year-old female presented with a five-month history of diarrhoea with watery stools seven times per day, nocturnal abdominal pain, asthenia, and a weight loss of 30% of her body mass in three months. The patient had a four-year medical history of bilateral mechanic gonalgia, arthralgia of the metacarpophalangeal and interphalangeal joints, and anaemia. The examination was remarkable for hyperpigmentation, along with a body mass index (BMI) of 15.8 kg/m^2^. The diagnosis of Whipple’s disease was made with upper gastrointestinal endoscopy, with typical histologic findings and a positive PCR for *Tropheryma whipplei*. Investigations also revealed cerebral demyelination and endocarditis in three valves. Treatment with intravenous ceftriaxone for four weeks and oral cotrimoxazole for one year resulted in complete resolution of the symptoms and endocarditis. This case report shows uncommon features of the disease and debates the challenging decisions toward diagnosis and effective treatment.

## Introduction

Initially denominated "intestinal lipodystrophy" in 1907 by George H. Whipple, given the fact that he observed "fatty masses in the lymphatic spaces" [1], Whipple’s disease (WD) is caused by *Tropheryma whipplei* and is a rare condition with multi-organ involvement. Its global incidence is 30 cases per year [2]. The current standard of care is based on antibiotic treatment and symptomatic control.

## Case presentation

Patient information

A 49-year-old female, completely independent, who was born and lived in Portugal and worked as a kitchen manager, presented with a four-year medical history of mechanic bilateral gonalgia and arthralgia of the metacarpophalangeal and interphalangeal joints, with concomitant iron-deficiency anaemia. She had a diagnosis of rheumatoid factor-negative migratory polyarthritis from rheumatology; nonetheless, there was no symptom relief while treated with prednisolone and glucosamine. The patient had no other past medical issues, no allergies, recent travels, or animal contact.

The patient was admitted with a chief complaint of a five-month course of diarrhoea, with watery stools seven times per day with a brownish colour and no visible blood or mucous associated. These features had not subsided with loperamide. The patient also noted nocturnal abdominal pain, asthenia, and a weight loss of 30% of her body mass in three months (15 kg), with no fever or night sweats. When the patient was admitted, she had already had ciprofloxacin and iron supplementation with no clinical improvement.

Physical examination

The most evident clinical findings during observation in the emergency department were hyperpigmentation of the face and thorax, as well as a body mass index (BMI) of 15.8 kg/m^2^. The patient had generalized abdominal pain but no palpable adenopathy or organomegaly.

Timeline

**Figure 1:**
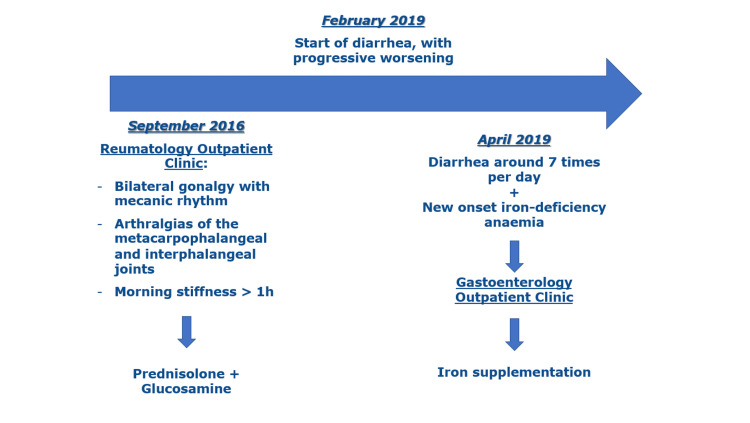
Timeline of the clinical findings and management before attending the emergency department.

A summary of the clinical findings and management of the patient's condition before being admitted to the ED in July 2019 is organised in a timeline (Figure 1).

Diagnostic assessment

During the initial diagnostic approach, there were laboratory findings of microcytic hypochromic anaemia, with haemoglobin 8.2 g/dL, erythrocyte sedimentation rate of 29 mm/h, no leucocytosis or neutrophilia, and a CRP of 4.34 mg/dL. An abdominal CT scan showed evidence of diffuse distention of the colon, with liquid content all along the jejune and multiple ganglia images. These ganglia images were remarkable for their distribution in retroperitoneal and mesenteric locations and their lipomatous content. A diagnosis of tuberculosis, lymphoproliferative disease, common infectious causes of diarrhoea, inflammatory bowel disease, celiac disease, and malabsorption syndrome was ruled out.

**Figure 2:**
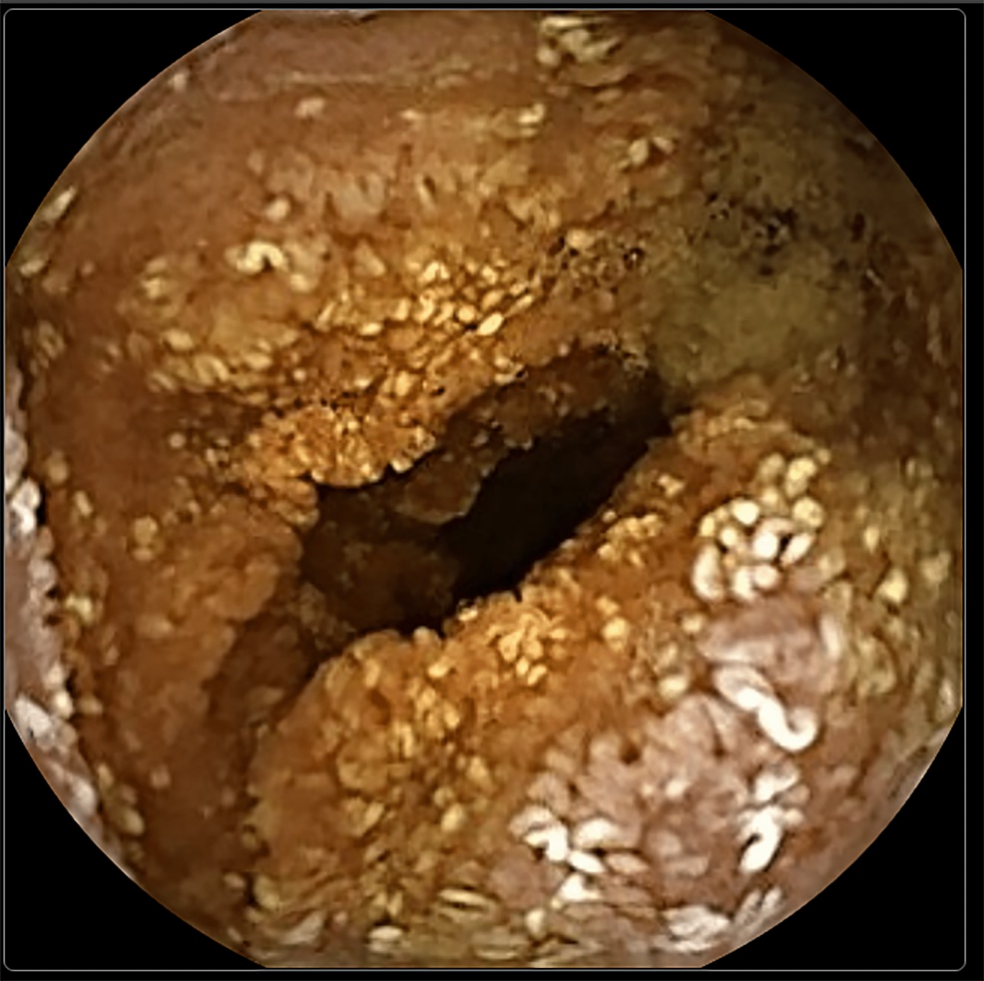
Image from capsule endoscopy, with evidence of villous atrophy and lymphangiectasia, macroscopically characteristic of Whipple's disease.

**Figure 3:**
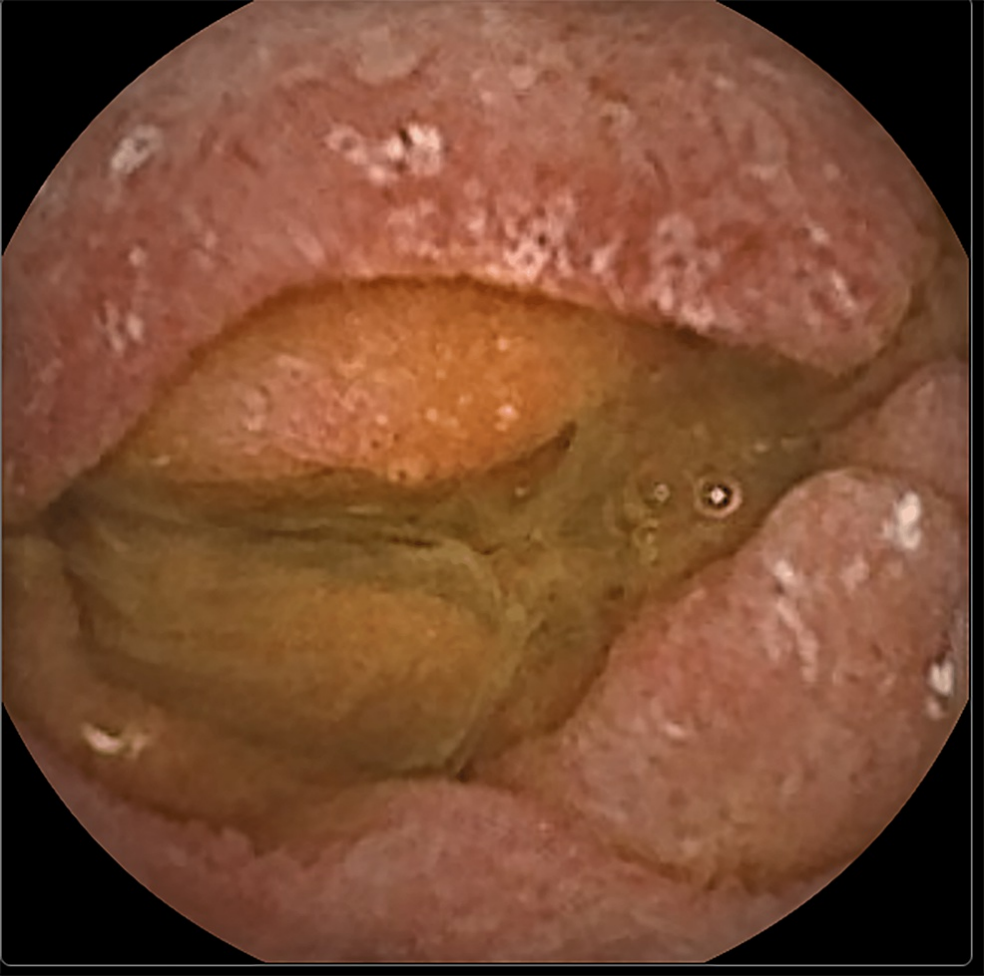
Image from capsule endoscopy, with evidence of oedema and erosions, macroscopically characteristic of Whipple's disease.

Since the upper GI endoscopy and colonoscopy revealed no macroscopic abnormalities, the patient had a capsule endoscopy, which revealed the macroscopic characteristics of Whipple's disease (Figures 2-3). Enteroscopy was performed to collect material from the duodenum (D2 and D3).

The patient's condition had clinically worsened in the few days before the enteroscopy, with severe asthenia, worsened diarrhoea, and anaemia necessitating blood transfusions and intravenous iron supplementation. Therefore, she was started on ceftriaxone given the highly expected diagnosis of WD.

**Figure 4:**
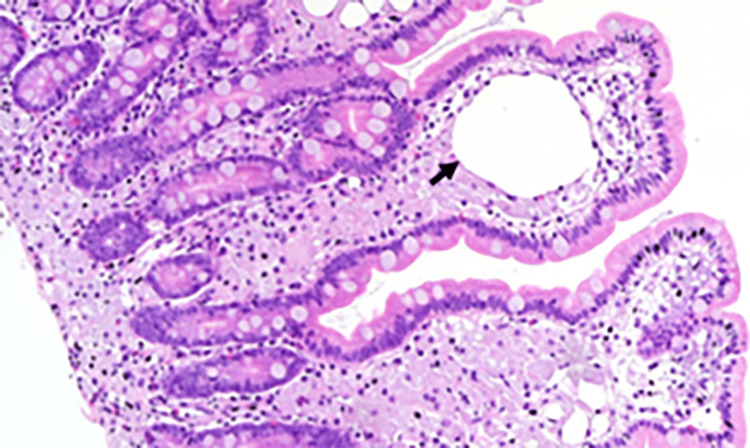
HE, 100×. The duodenal mucosa showed a diffuse infiltration of the lamina propria and submucosa by foamy macrophages that blunted and distended the villi. Lymphangiectasia was also seen (arrow).

**Figure 5:**
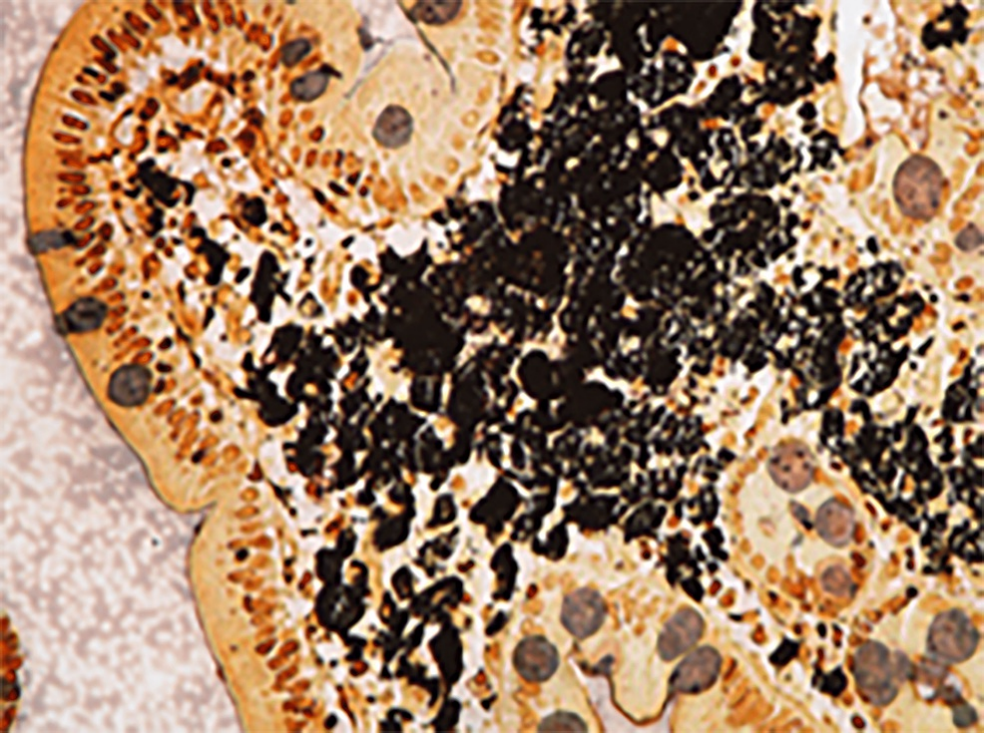
Warthin-Starry, 200×. The foamy macrophages showed intense staining, with a Warthin-Starry stain, of the intracellular material that gives their cytoplasm their characteristic granularity.

Specimens from D2 had negative Ziehl-Neelsen staining and mycobacterial culture, but the DNA-PCR for *Tropheryma whipplei* was highly positive and positive in the blood sample, confirming the diagnosis. The histology also showed the typical finding of macrophages containing diastase-resistant PAS-positive granules (Figures 4-5).

**Figure 6:**
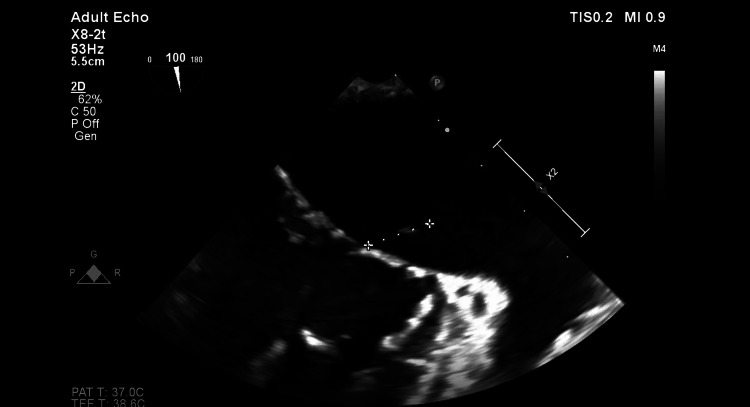
Image from echocardiogram, with evidence of endocarditis of the mitral valve.

**Figure 7:**
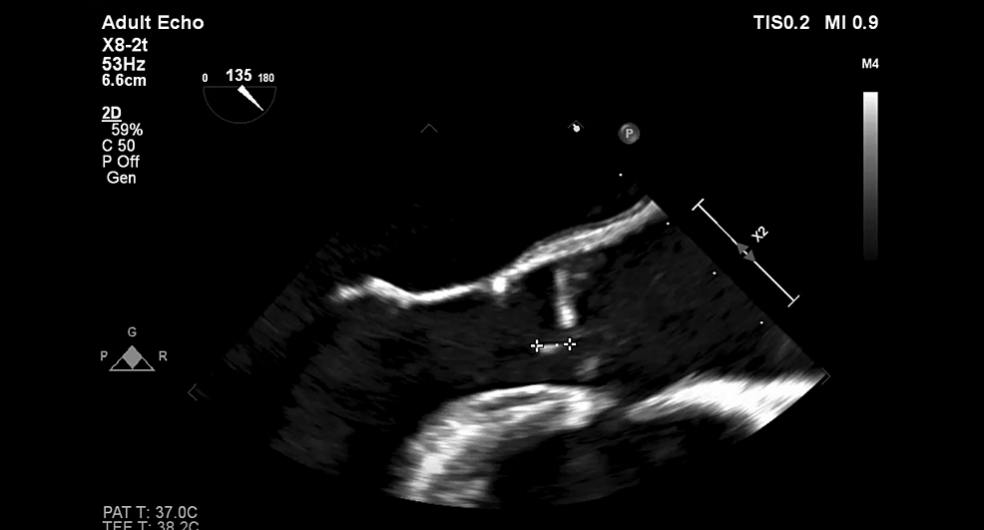
Image from echocardiogram, with evidence of endocarditis of the aortic valve.

Given this diagnosis, and even though the neurological exam was normal, a brain MRI was performed and showed areas of unspecific demyelination. Furthermore, given the fact that WD can be associated with negative blood culture endocarditis, even though there were not any heart-related symptoms since the presentation, the patient had a transoesophageal echocardiogram. This showed preserved systolic function (EF 57%), with vegetations seen in the mitral, tricuspid, and aortic valves (Figures 6-7).

Interventions and follow-up

The patient showed clinical improvement after three days of antibiotics, with prompt resolution of the diarrhoea. The patient completed four weeks of intravenous ceftriaxone, followed by one year of oral TMP-SMP. The patient was also put on a progressive oral protein and calories oral diet.

After discharge, the patient had a follow-up in our internal medicine outpatient clinic, with the recuperation of the usual weight after six months. The antibiotic treatment prescription was given every two months to maintain adherence surveillance. The patient had total tolerability of the antibiotic regimen.

## Discussion

The classical clinical presentation of WD starts with polyarthralgia, with bowel involvement occurring approximately after four years of illness. WD is frequently underdiagnosed and should be considered in all patients with the four cardinal manifestations: arthralgias, diarrhoea, abdominal pain, and weight loss. An additional feature is skin hyperpigmentation, reported in 40-45% of cases [3].

The response to treatment can be monitored by haemoglobin and haematocrit, weight, and symptom resolution. One month after discharge, the patient had haemoglobin 12.2 g/dL, no iron deficiency, and a negative faecal occult blood test. Ten months after starting treatment, a transoesophageal echocardiogram showed complete resolution of the endocarditis. During treatment, there was also a resolution of the characteristic hyperpigmentation, no recurrence of diarrhoea and fever, and a resolution of arthralgias. The patient maintained no signs of heart failure or any focal neurological signs in the next three years of follow-up.

WD should be a consideration in patients with rheumatoid factor-negative migratory polyarthritis that does not respond to immunosuppressive therapy, which was the case of the patient reported. The wide range of clinical features makes this a challenging diagnosis, causing disease in multiple organs and systems, namely the bowels, central nervous system, heart, lungs, and uvea. WD is increasingly recognized as an important cause of culture-negative endocarditis, even though the involvement of multiple valves as in this case is rarely reported [4].

WD was uniformly fatal prior to the availability of antibiotics but can be successfully treated with them, although the optimal regimen is still uncertain. Clinical relapses have been reported in 17-35% of patients [3], reflecting the importance of follow-up.

## Conclusions

The most important limitation in diagnosing and treating patients with WD is that it is a rare disease. There are still no guidelines for the assessment and treatment of these patients, with capital importance on the literature review and clinical appreciation needed to decide which antibiotic regimen to choose and the duration of treatment.

This case report shows the prognostic significance of the correct identification of WD as a cause of unintentional weight loss associated with arthralgias and diarrhoea. It is important to note that, when not recognized and properly treated, Whipple’s disease is fatal. Further investigation is important to understand the risk factors for this disease, such as host immune deficiency and secondary immune downregulation induced by the bacterium.
